# Lack of association between mannose-binding lectin gene polymorphisms and juvenile idiopathic arthritis in a Han population from the Hubei province of China

**DOI:** 10.1186/ar1953

**Published:** 2006-05-08

**Authors:** Min Kang, Hong-Wei Wang, Pei-Xuan Cheng, Zun-Dong Yin, Xiao-Ou Li, Hong Shi, Xiu-Fen Hu

**Affiliations:** 1 Department of Pediatrics, Tongji Hospital, Tongji Medical College, Hua Zhong University of Science and Technology, 1095 Jie Fang Avenue, Wuhan 430030, Hubei Province, China; 2 Chinese Center for Disease Control and Prevention, 27 Nan Wei Road, Beijing 100050, China

## Abstract

Many studies have reported that polymorphisms of the mannose-binding lectin (MBL) gene are associated with autoimmune disease. Here, we investigate the relationship between MBL gene polymorphisms and susceptibility to juvenile idiopathic arthritis (JIA) in a Han-nationality population from the Hubei province of China. PCR-restriction fragment length polymorphism was used to investigate polymorphisms of codons 54 and 57 in exon 1 of the MBL gene in 93 patients with JIA and 48 control children. Neither group showed codon 57 polymorphisms. There was no significant difference in the genotypic frequencies of codon 54 between patients with JIA and healthy controls (wild type, 71.0% versus 75.0%, respectively; heterozygous type, 25.8% versus 25.0%, respectively; and homozygous type, 3.2% versus 0.0%, respectively). In addition, no association was found between the subgroups of patients with JIA and control individuals. Our results provide no evidence for a relationship between MBL gene mutation and susceptibility to JIA.

## Introduction

Mannose-binding lectin (MBL) is an important component of host innate immunity, which has a nonspecific role in complement activation and opsonization. There are three single-point mutations that have been well characterized in exon 1 of the MBL gene, at codon 52 (CGT→TGT), codon 54 (GGC→GAC) and codon 57 (GGA→GAA), and these differ considerably in their frequencies in different populations [1,2]. MBL gene polymorphisms were reported to have an important role in regulating both the serum MBL level and MBL activation. Several publications have suggested that a low serum level of MBL in humans is associated with recurrent infection [3,4]. Moreover, there is evidence that MBL mutation or deficiency is an additive risk factor for susceptibility to autoimmune disease, such as systemic lupus erythematosus and rheumatoid arthritis [5-7].

Juvenile idiopathic arthritis (JIA), formerly known as juvenile rheumatoid arthritis, is the most common pediatric autoimmune disease, with a high incidence of disability [8]. JIA is both similar and distinct from adult-onset arthritis [9]. This article summarizes the relationship between MBL gene polymorphisms and susceptibility to JIA.

## Materials and methods

### Patients and controls

The subjects enrolled in this study included 93 patients with JIA and 48 healthy children. All patients were diagnosed according to the International League of Associations for Rheumatology (ILAR) classification criteria for JIA [8]. According to the ILAR criteria, the patients with JIA in our study were divided into five subgroups: 26 patients with systemic-onset JIA, 23 patients with rheumatoid factor (RF)-negative polyarthritis, 15 patients with RF-positive polyarthritis, 16 patients with oligoarthritis and 13 patients with enthesitis-related arthritis. The mean age of patients with JIA was 8.5 years (range, 2–15 years) and the mean disease duration was 26.2 months (range, 7–60 months). The gender distributions in the group of patients with JIA and the control group were not significantly different. All the subjects were Han-nationality Chinese from the Pediatric Department of Tongji Hospital, Tongji Medical College, Huazhong University of Science and Technology in Wuhan City, Hubei province, China. Participation was voluntary.

### Detection of mannose-binding lectin gene polymorphisms

Polymorphisms in codons 54 and 57 of the MBL gene were analyzed by PCR-restriction fragment length polymorphism (PCR-RFLP). Briefly, for determination of polymorphisms in codons 54 and 57, a fragment of 315 base pairs (bp) was amplified using the following primers: 5'-ATAGCCTGCACCCAGATTGTAG-3' (forward primer) and 5'-AGAGACAGAACAGCCCAACAC-3' (reverse primer). The PCRs were performed in a final volume of 25 μl using 2.5 mM MgCl_2_, 2.5 mM for each deoxyribonucleotide triphosphate (dNTP) and 5 U/μl Ampli *Taq *DNA polymerase (TaKaRa Biotechnology(Dalian)Co., Ltd, Shiga, Japan). PCR conditions were as follows: an initial denaturation at 94°C for 5 minutes; 34 cycles of denaturation at 94°C for 30 seconds, annealing at 60°C for 1 minute and extension at 72°C for 1 minute; and a final elongation at 72°C for 5 minutes. The PCR products were digested with the restriction enzymes *Ban*I and *Mbo*II for codons 54 and 57, respectively. The genotypes were determined by electrophoresis on 2% agarose gels stained with ethidium bromide. For codon 54, a fragment with a wild-type allele was cleaved into two bands (of 263 bp and 52 bp), whereas the fragment with a mutant allele showed one band (of 315 bp). For codon 57, a fragment with a wild-type allele showed one band (of 315 bp), whereas the fragment with a mutant allele was cleaved into two bands (of 272 bp and 43 bp).

### Statistical analysis

The chi-squared test and Fisher's exact test were used to calculate significant differences between patients and controls. An SPSS software package (version 11.0; SPSS Inc., Chicago, IL, USA) was used to analyze the data.

## Results

### Findings in healthy controls

The distribution of codon 54 gene polymorphisms in healthy Han-nationality Chinese children is shown in Table 1. The genotype and allele A frequencies of codon 54 did not show significant differences compared with those of healthy controls from Hong Kong and Europe [6,7]. The mutation type of codon 54 was seen in 25.0% of Chinese children (*n* = 48), 21.4% of adults from Hong Kong (*n *= 196) and 24.6% of European adults (*n *= 114).

### Findings in patients with juvenile idiopathic arthritis

No deviation from the Hardy-Weinberg equilibrium (HWE) was detected in patients with JIA or healthy control individuals (Table 2). The frequency of the mutation type was higher in patients with JIA than controls, but was not significantly different.

#### Codon 54 mutations in subgroups of patients with juvenile idiopathic arthritis

Table 3 shows genotypic and allelic frequencies of codon 54 in the subgroups of patients with JIA. The heterozygous type was observed in all the subgroups of patients with JIA, including 6 patients with systemic-onset JIA, 6 patients with RF-negative polyarthritis, 5 patients with RF-positive polyarthritis, 3 patients with oligarthritis and 4 patients with enthesitis-related arthritis. In addition, three homozygous types were found: two in patients with systemic-onset JIA and one in a patient with RF-positive polyarthritis.

#### Analysis of the genotype of codon 54 and serum indices in patients with juvenile idiopathic arthritis

The relationship between the number of patients who had codon 54 mutations and both RF seropositivity and antinuclear antibody (ANA) seropositivity is shown in Table 4. There were no significant differences in the number of patients with either heterozygous or homozygous codon 54 mutations between patients with JIA who were positive or negative for RF and ANA.

Our study did not find polymorphism in codon 57 of the MBL gene.

## Discussion

MBL is one of the most important constituents of the innate immune system [10,11]. Many studies have reported a deficiency of MBL in different populations [1]. This is the first investigation of an association between MBL polymorphisms and JIA. Our study showed the genotypic and allelic frequencies in codon 54 were consistent with previous reports from Hong Kong and Europe [6,7]. Our study did not find a codon 57 mutation. Similar results were reported in Japanese and Korean populations, although codon 57 mutations were found in 50–60% of African populations [12,13]. The different distribution of genotypic and allelic frequencies in different populations might exist as a result of ethnic difference, in addition to migration [14].

JIA is a common autoimmune disease, and infection has a role in its etiology [15,16]. We recognize that the enhanced risk of the development of recurrent infection, in addition to systemic lupus erythematosus triggered by complement pathway deficiency states, indicates the importance of the complement system in the protection against disease [5,17]. Studies have shown that the MBL pathway is an independent pathway of complement activation [2]. Mutation and deficiency of the MBL codon could impair the ability of pathogen or immune-complex clearance, facilitating the development of autoimmunity [17,18]. Therefore, we hypothesize that opsonization dysfunction of the complement system caused by MBL gene mutation is involved in the immune response to infection. However, the polymorphism of MBL did not seem to be associated with JIA or any of its subgroups in our study. In addition, no significant differences were found in the number of patients with heterozygous or homozygous mutations of codon 54 in patients with JIA who were positive or negative for RF or ANA.

JIA represents a heterogeneous group of chronic arthritis in children, with variable presentations and courses. Although the etiology of JIA is still unknown, the involvement of diverse HLA-DRB1 alleles (such as DRB1*03, DRB1*04, DRB1*08, DRB1*09, DRB1*15) has been well established by many reports [19,20]. Moreover, it is thought that MBL deficiency is not an independent trigger for infection and autoimmune disease. MBL polymorphisms are supposed to have a synergistic effect on the susceptibility to disease [21,22]. Our study seems to rule out a direct association between MBL gene polymorphisms and JIA. Because this study included a small number of subjects, further research in large cohorts of patients should be carried out to reach a conclusion.

## Conclusion

The association of MBL polymorphism with JIA requires further investigation. This study provides no evidence for MBL gene mutation with respect to JIA. However, MBL gene mutation might have a role in the early diagnosis of the subgroups of JIA.

## Abbreviations

ANA = antinuclear antibody; bp = base pairs; dNTP = deoxyribonucleotide triphosphate; HWE = Hardy-Weinberg equilibrium; ILAR = International League of Associations for Rheumatology; JIA = juvenile idiopathic arthritis; MBL = mannose-binding lectin; PCR = polymerase chain reaction; PCR-RFLP = PCR-restriction fragment length polymorphism; RF = rheumatoid factor.

## Competing interests

The authors declare that they have no competing interests.

## Authors' contributions

MK prepared the manuscript, which was modified by HWW. HWW designed the study. MK and XOL carried out molecular genetic studies. PXC, HS and XFH contributed to the acquisition of clinical data. ZDY contributed to the statistical analyses.

## Figures and Tables

**Table 1:** Genotypic and allelic frequencies of codon 54 mutations in healthy controls

	Healthy control, *n *(%)	Hong Kong control^a^, *n *(%)	European control^b^**, ***n *(%)
Wide type	36 (75.0)	153 (78.1)	84 (73.7)
Heterozygous type	12 (25.0)	42 (21.4)	28 (24.6)
Homozygous type	0 (0.0)	1 (0.5)	2 (1.7)
Frequency of allele A	0.12	0.11	0.14

^a^Data from [6].^b^Data from [7].

**Table 2:** Genotypic and allelic frequencies of codon 54 mutations in patients with JIA and control children

Groups	Genotype			Allele		HWE *P *value
		
	Wild type	Heterozygous type	Homozygous type	G	A	
JIA, *n* (%)	66 (71.0)	24 (25.8)	3 (3.2)	0.84	0.16	0.66
Control, *n* (%)	36 (75.0)	12 (25.0)	0 (0.0)	0.88	0.12	0.32
*P*		0.69^a^		0.42^b^		

^a^Fisher's exact test. ^b^Chi-squared test. HWE, Hardy-Weinberg equilibrium; JIA, juvenile idiopathic arthritis.

**Table 3:** Genotypic and allelic frequencies of codon 54 mutations in subgroups of patients with JIA

Subtype of JIA	Genotype			Allele	
	
	Wild type	Heterozygous type	Homozygous type	G	A
Systemic onset, *n *(%)	18 (69.2)	6 (23.1)	2 (7.7)	0.808	0.192
RF(-) polyarthritis, *n *(%)	17 (73.9)	6 (26.1)	0 (0.0)	0.870	0.130
RF(+) polyarthritis, *n *(%)	9 (60.0)	5 (33.3)	1 (6.7)	0.767	0.233
Oligoarthritis, *n *(%)	13 (81.2)	3 (18.8)	0 (0.0)	0.906	0.094
ERA, *n *(%)	9 (69.2)	4 (30.8)	0 (0.0)	0.846	0.154
*P *value		0.84^a^		0.57^b^	

^a^Fisher's exact test. ^b^Chi-squared test. ERA, enthesitis-related arthritis; JIA, juvenile idiopathic arthritis; RF(-), RF-negative; RF(+), RF-positive.

**Table 4:** Codon 54 mutations of MBL and serological indices in patients with JIA

Indices		JIA genotype (*n *= 93)		*P *value
			
		Wild type (*n *= 66)	Mutation type (*n *= 27)	
RF	positive (*n *= 15) negative (*n *= 78)	9 57	6 21	0.36
ANA	positive (*n *= 16) negative (*n *= 77)	11 55	5 22	1.00

ANA, antinuclear antibody; JIA, juvenile idiopathic arthritis; RF, rheumatoid factor.
